# Towards Green and Sustainable Healthcare: A Literature Review and Research Agenda for Green Leadership in the Healthcare Sector

**DOI:** 10.3390/ijerph20020908

**Published:** 2023-01-04

**Authors:** Justyna Berniak-Woźny, Małgorzata Rataj

**Affiliations:** 1 Department of Management, University of Information Technology and Management, 35-225 Rzeszów, Poland; 2 Department of Cognitive Science and Mathematical Modeling, University of Information Technology and Management, 35-225 Rzeszow, Poland

**Keywords:** green energy, renewable energy, innovation, PRISMA, bibliometric analysis

## Abstract

The health sector is one of the keys to sustainable development. Although it is directly related to only one Sustainable Development Goal (Goal 3, “Ensuring a healthy life and promoting well-being at all ages”), the sector itself, which aims to protect health, is paradoxically at the same time the main emitter of environmental pollutants that have a negative impact on health itself. Therefore, sustainability has become a key priority for health sector organizations, and leadership in this area is essential at all levels. Scientific research plays a particular role here, helping to more clearly define the links between environmental sustainability and the health effects of a polluted environment and climate change as well as indicating the direction of actions needed and disseminating good practices that can help accelerate the adoption of efforts towards climate neutrality and sustainable development of health sector organizations. The aim of this article is to present the current state of the art and future research scenarios in the field of green and sustainable healthcare through a literature review by using the Preferred Reporting Items for Systematic Reviews Meta-Analyses (PRISMA) method to perform a bibliometric analysis of papers published in 2012–2022. The Web of Science Core Collection (WoSCC) database is used for this purpose. A total of 144 papers are included for analysis, categorized based on eight fields: author(s), title, year of publication, country, journal, scientific category, and number of citations. Based on the results, themes for future research on green leadership in the healthcare sector are identified and recommended.

## 1. Introduction

Sustainable development is one of the greatest, if not the greatest, global challenges of the 21st Century. However, the importance of sustainable development had already been recognized and declared as early as the United Nations Conference on the Human Environment in Stockholm in 1972 [1]. Fifteen years later, sustainable development was defined by Gro Harlem Brundtland, former Norwegian Prime Minister and Chair of the World Commission on Environment and Development (WCED), as “meeting the needs of the present without compromising the ability of future generations to meet their own needs” [2]. In 1992, the United Nations organized the Earth Summit in Rio de Janeiro, during which one of the most important documents related to sustainable development was prepared, Agenda 21, a comprehensive plan of action for the 21st Century for the United Nations, governments, and social groups in every area in which man has an impact on the environment. The Earth Summit was attended by representatives of 172 governments, 2400 NGOs, and 10,000 journalists, and 172 countries signed the Agenda [3]. The document includes the statement that humanity has reached a turning point in history, and a cautionary tale that by continuing the present policy we contribute to the widening of the economic gap in societies and between countries, the expansion of poverty, hunger, disease, and illiteracy. We cause a progressive degradation of the natural environment on which life on Earth depends [4]. Additionally, a proposal to change procedures in the future was defined. Recommendations range from new teaching methods to new methods of using raw materials and contributing to a sustainable economy. The overall ambition of Agenda 21 is a safe and just world in which every living thing is able to maintain its dignity. In parallel, the United Nations has launched a number of initiatives for sustainable development. The first comprised the Millennium Development Goals (MDGs), including eight interrelated goals as a holistic process, set at the 2000 Millennium Summit for a period of 15 years to 2015 [5]. Given its achievements and importance, in 2015 the United Nations established the 2030 Agenda for Sustainable Development, agreed to by 195 countries and consisting of 17 goals and 169 measures related to economic, environmental, and social objectives [6,7,8].

Sustainable development is one of the key issues in the healthcare industry. While of the 17 Sustainable Development Goals (SDGs) only the third goal of “ensuring healthy living and promoting well-being for all ages” is directly relevant to the health sector, other SDGs (such as on hunger, gender equality, clean water and sanitation, affordable and clean energy, sustainable cities and communities, climate action, peace, justice and strong institutions) with 43 health-related indicators apply to this industry indirectly. Even though the last two decades have been called a golden age for global health due to the increase in national health spending and donor funding by low- and middle-income countries, which has translated into increased access to health determinants (such as clean water and sanitation) and health services (such as vaccination, antenatal care, and HIV treatment) [9], recent years have only seen improvements in 24 (56%) of the 43 health-related SDG indicators, as WHO data shows [10].

However, satisfying the health needs of the population is associated with a negative impact on the natural environment, as health care is one of the main emitters of environmental pollution that has a negative impact on health. In Brazil alone, hospitals consume 10.6% of the energy used for commercial purposes [11]. In the UK, the National Health Service (NHS) emits 18 million tonnes of CO_2_ annually, accounting for almost a quarter of the total emissions coming from the public sector [12]. In the US, total gas emissions from healthcare organizations increased by 6% from 2010 to 2018 [13,14]. India generated over 33,000 tonnes of medical waste during the seven months of the COVID-19 pandemic [15]. Healthcare, including pharmaceuticals, is responsible for 4.4% of global greenhouse gas emissions globally. In addition, the global market for medical waste management is expected to grow from an estimated USD 6.8 billion in 2020 to USD 9 billion by 2025 [16]. This negative impact was further exacerbated during the COVID-19 pandemic, mainly as a result of the increased intensity of health sector activities and the increased use of personal protective equipment (PPE) as well as diagnostic tools and vaccines for severe acute respiratory syndrome coronavirus 2 (SARS-CoV-2), which in both cases translated into an increase in the generation of medical waste. [17] Thus, the healthcare sector strives to transform itself into a sustainable one.

Sustainable healthcare can be defined as suggested - “a complex system of interacting approaches to the restoration, management and optimisation of human health that has an ecological base, that is environmentally, economically and socially viable indefinitely, that functions harmoniously both with the human body and the non-human environment, and which does not result in unfair or disproportionate impacts on any significant contributory element of the healthcare system” [18]. Sustainable healthcare is often referred to as green healthcare, which means the provision of healthcare services in an environmentally friendly manner that aims to promote health while having a positive impact on the community [19,20]. The concept of green hospitals defined by Howard in a report of the US Office of the Federal Environmental Executive is popular in this regard, and is defined as the practice of increasing the efficiency with which buildings and their sites use energy, water, and materials while reducing building impacts on human health and the environment through better siting, design, construction, operation, maintenance, and removal. [21] Indeed, according to Kreisberg [22], green healthcare facilitates a sustainable future for medicine, physicians, patients, and the environment. According to Fadda [19], green health systems are based on the following ten components.

(1) Leadership through education, goal setting, accountability, and incorporating these priorities in all external relations and communications
(2) Substituting harmful chemicals with safer alternatives
(3) Reducing, treating, and safely disposing of healthcare wastes
(4) Implementing energy efficiency and clean renewable energy generation
(5) Reducing hospital water consumption and supplying potable water
(6) Improving transportation strategies for patients and staff
(7) Reducing food waste and the environmental footprint while improving patient and worker health by making changes in hospital service menus and practices
(8) Reducing pharmaceutical pollution and developing safer pharma
(9) Taking advantage of green buildings to develop safer, more resilient, greener, and healthier building products and systems
(10) Changing purchasing habits in ways that reduce environmental and human rights impacts.

Unfortunately, the awareness of the healthcare community regarding the negative impact of the sector on the natural environment and society, and in turn the responsibility for dealing with it, is very low [23]. To support sustainable health systems, leadership is essential at all levels [24,25,26,27], both at the level of green politics [28,29,30] and at the level of influencing and shaping the attitudes of members of the health community and organizations [31]. Fortunately, in recent years there have been promising examples of green leadership at the international level, such as by the following organizations:

- The Alliance of Nurses for Healthy Environments (ANHE), a nursing organization focused solely on the intersection of health and the environment [32].
- Health Care Without Harm (HCWH), an international nongovernmental organization (NGO) that works to transform health care worldwide to ensure that it reduces its environmental footprint and becomes a community anchor for sustainability and leader in the global movement for environmental health and justice [33].
- The Health and Environment Alliance (HEAL), the leading European not-for-profit organisation addressing how the natural and built environments affect health in the European Union (EU) and beyond [34].
- The Global Climate and Health Alliance, made up of health and development organizations from around the world united by a shared vision of an equitable and sustainable future. Alliance members work together to (1) ensure that health impacts are integrated into global, regional, national, and local policy responses to climate change to reduce them as far as possible, with a particular focus on reducing health inequalities through mitigation and adaptation; (2) encourage and support the health sector to lead by example in mitigating and adapting to climate change; (3) raise awareness of the health threats posed by climate change and the potential health benefits of well-chosen climate mitigation policies in areas such as energy, transport, food, and housing [35].
- Irish Doctors for Environment is an NGO and registered charity consisting of doctors, medical students, and allied healthcare professionals in Ireland who aim to create awareness and interest and implement action around environment health and the impact it has on patient health [36]
- OraTaiao: The New Zealand Climate and Health Council comprises health professionals in Aotearoa/New Zealand concerned with (1) the negative impacts of climate change on health; (2) the health gains that are possible through strong health-centred climate action; (3) highlighting the impacts of climate change on those who already experience disadvantage or ill-health (equity impacts); and (4) reducing the health sector’s contribution to climate change [37]
- The Canadian Association of Physicians for the Environment, which takes action to enable health for all by engaging with governments, running campaigns, conducting research, and drawing media attention to key issues [38]
- Doctors for the Environment Australia (DEA), an organisation of medical professionals that protect human health through care of the environment. The devastating impacts of climate change on human health and the solutions needed to address this grave threat are a major focus of their work. DEA members include GPs, surgeons, physicians, anesthetists, psychiatrists, pediatricians, public health specialists, academics, medical students, and researchers, bringing leadership and expertise from every branch of medicine [39].

To provide better guidance for the development and adoption of new practices and procedures in the field of sustainable and green healthcare, the aim of this article is to present the current state of the art and future research scenarios in the field of green and sustainable healthcare.

The rest of this paper is organized as follows. Section 2 defines the methodology and datasets. In Section 3, the main results of the review are presented and discussed. Finally, our conclusions are outlined, including the implications and limitations of the paper and future research directions. 

## 2. Materials and Methods

As mentioned in the Introduction, the aim of this article is to present the current state of the art and future research scenarios in the field of green and sustainable healthcare. To achieve the assumed goal, the authors conducted a review of the literature on green and sustainable healthcare published in 2013–2023 (early access) using the Preferred Reporting Items for Systematic Reviews and Meta-Analyses (PRISMA) guidelines [40]. The review was supplemented with a bibliographic analysis to analyze large numbers of publications and identify research trends and patterns in the defined research area [41]. This allowed for a holistic view of the constantly growing knowledge resources and the assessment of specific research directions as well as the outlining of the anatomy of current knowledge in a green and sustainable healthcare field [42]. 

The search was carried out in October 2022. The search process used the Web of Science (WoS) Core Collection database, which is the leading database for classifying academic research. The Web of Science Core Collection contains over 21,100 peer-reviewed, high-quality scholarly journals published worldwide in over 250 scientific disciplines. Conference proceedings and book data are available as well. The WoS Core Collection was analyzed to find related publications based on the following keyword combination: “sustainability” OR “green” AND “healthcare/health care”. We searched for articles with these phrases in the title, abstract, or keywords. Additionally, the search was limited to records published from 2013 to 2022 in English. The results of these searches contributed to the selection of a database consisting of 4289 documents that matched our query. The WoS database was downloaded as a file in PDF format. Further, we screened the titles and abstracts, which limited the database to 836 records. The selection process involved two independent reviewers and two steps: (1) selection based on inclusion criteria (publications on sustainable and/or green healthcare systems or institutions and publications with a minimum of ten citations) and (2) final inclusion in the review. Discrepancies between reviewers were resolved through discussion and agreement. The database was downloaded in the TXT format, as the authors planned to use it for visualization in VOSviewer software, which requires CSV or TXT files. As WOS has built-in analyzer features, initial descriptive analysis was carried out using these features, and further Excel analyzing features were employed. Tables were created to provide quantitative data. Additionally, VOSviewer software version 1.6.18 was applied to quantitatively and visually analyze keyword co-occurrences.

**Figure 1:**
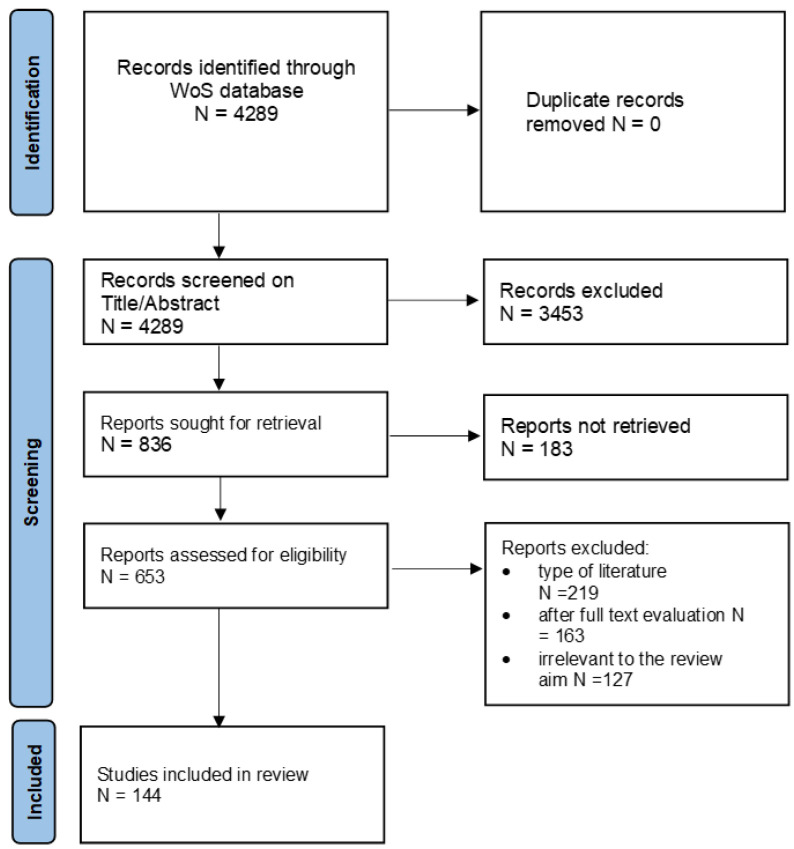
PRISMA flow chart [43].

Next, as we wanted to have access to the full content of the articles, the database was narrowed to 653 records. Further, in order to focus on scientific contributions and avoid editorials and other unrelated material, reviews, editorial materials, and notes were excluded. The database was narrowed down to articles and proceedings only, and 219 records were excluded. The analysis of the full text of the publications resulted in the exclusion of a subsequent 163 records. The next round of full text analysis eliminated a further 127 records, as they were considered to be irrelevant to the review aim or were not published in a journal with sufficient impact factor. As shown in Figure 1, the final database consisted of 144 documents, including 142 articles and two proceedings papers. 

## 3. Results

To provide a holistic academic landscape and understandable overview of the latest trends in research related to green and sustainable healthcare, the results of the review are presented from the perspective of (1) the number of publications between 2013–2022 and the keywords clusters, (2) leading countries, (3) leading journals, (4) the most impactful papers, and (5) major disciplines. 

### 3.1. Results—Total Number of Publications

**Figure 2:**
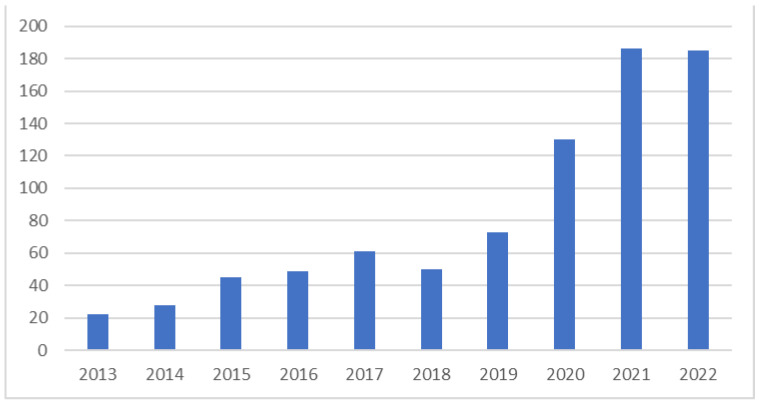
Number of articles published in the years 2013–2022.

The number of publications over time, or the growth trend, is one of the most relevant factors as to how much scholars are interested in a specific topic, and is an indicator of the expansion of a field of research [44,45,46]. Figure 2 shows the yearly distribution of the selected articles on green and sustainable healthcare in the last decade. The growth in the annual number of published articles in 2020–2022 reflects the growing popularity of the subject around the world.

The significant increase in the last three analyzed years is interesting. These dynamics of publication growth in the area of a sustainable and green healthcare may be related to the crisis caused by the pandemic. Examples of the articles on the issues analyzed from the perspective of COVID are as follows:

- “Implementation of Obstetric Telehealth During COVID-19 and Beyond” [47],
- “Framework for PESTEL dimensions of sustainable healthcare waste management: Learnings from COVID-19 outbreak” [48],
- “Selection of the best healthcare waste disposal techniques during and post COVID-19 pandemic era” [49],
- “Leveraging nurse practitioner capacities to achieve global health for all: COVID-19 and beyond” [50],
- “Development of a Multi-Criteria Model for Sustainable Reorganization of a Healthcare System in an Emergency Situation Caused by the COVID-19 Pandemic” [51]
- “How Can Health Systems Better Prepare for the Next Pandemic? Lessons Learned from the Management of COVID-19 in Quebec (Canada)” [52].

### 3.2. Results—Keyword Analysis

**Figure 3:**
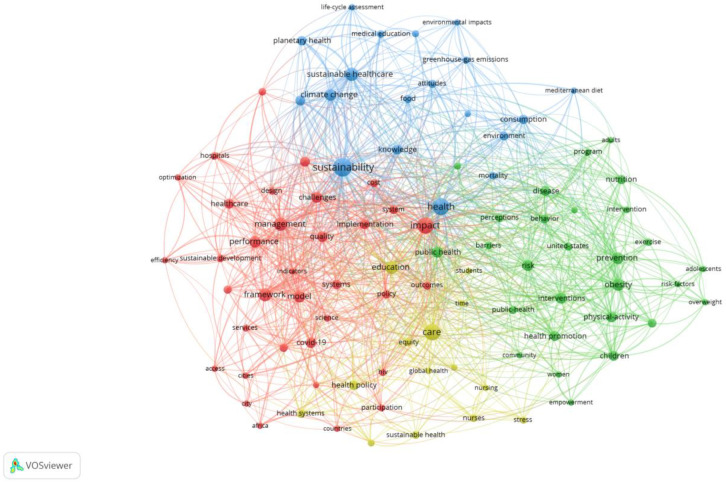
Keywords co-occurrence map.

The keyword analysis began with the initial sample of 836 publications, on which mapping was performed based on VOSviewer software. Figure 3 shows a graphical representation of keyword co-occurrences. Only keywords which appeared at least ten times in our sample were covered by the analysis. Keywords that occurred more frequently are represented with a larger font size and circle. Keywords that appeared together are linked with lines. A thicker line between two keywords indicates that these two keywords appeared together more often in one publication. Looking at Figure 3, four different thematic clusters can be seen, represented by different colors.

First, the red cluster is the medical branch of research, which deals with sustainable development of health management. Keywords such as “health system”, “health policy”, “quality”, “design”, and “optimalization” indicate that the focus is on understanding implementation of green scenario in the context of health care. 

The blue cluster focuses on the natural environment, and consists of keywords such as “sustainability”, “environment”, or “climate change”. The blue cluster is about education, with keywords such as “knowledge”, “medical education”, and “attitudes”. In most cases, the ultimate goal of the articles within the green cluster is about implementation of green solutions in medical institutions. 

The green cluster is driven generally by prevention and risk assessment in the context of eco-health. In this cluster, “behavior”, “perception”, and “empowerment” are among the keywords. Closely related to the green cluster is general health, with terms such as “overweight”, “physical activity”, and “nutrition”. 

The yellow cluster is sustainable development in medical care, underlying how important is education in this field both for students and medical personnel. 

Overall, the keyword “co-occurrence network” in Figure 3 underpins the multidisciplinary nature of the green and sustainable health sector. 

By conducting a comprehensive analysis of the Authors’ Keywords in the final database generated from WoS (144 papers), the following three thematic clusters were formed.

**Figure 4:**
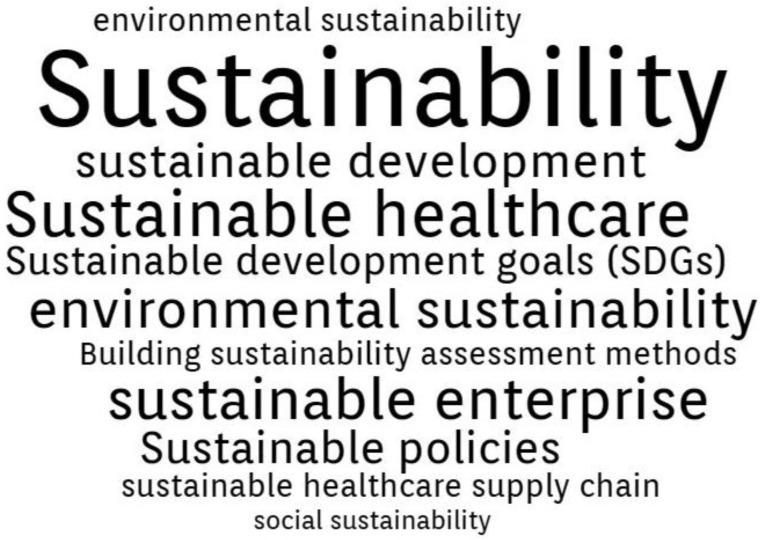
Sustainability cluster Authors’ Keywords.

Cluster 1—“Sustainability”. Presented in Figure 4, this cluster includes 20 terms: building sustainability assessment methods, environmental sustainability, sustainable development, sustainable employability, sustainable enterprise, sustainable healthcare, sustainable healthcare supply chain, sustainable healthcare systems, sustainable transportation, environmental sustainability, social sustainability, sustainable behaviors, sustainable business models, sustainable competitive advantage, sustainable design, sustainable development goals (SDGs), sustainable diets, sustainable physical healthcare pattern recognition, sustainable policies, sustainable health care education. In general, the papers referring to this cluster focus on:

- The 2030 Agenda of United Nations for Sustainable Development Goals (SDGs), for example, “Sustainable development goals and mental health: learnings from the contribution of the FundaMentalSDG global initiative” [53], “Soft power and global health: the sustainable development goals (SDGs) era health agendas of the G7, G20 and BRICS” [54], “Approaches to protect and maintain health care services in armed conflict—meeting SDGs 3 and 16” [55].
- Sustainability assessment methods and competitive advantage: “Assessment of Environmental Sustainability in Health Care Organizations” [56], “Advanced therapy medicinal products and health technology assessment principles and practices for value-based and sustainable healthcare” [57], “Development of a healthcare building sustainability assessment method—Proposed structure and system of weights for the Portuguese context” [58].
- Sustainable healthcare organization and systems: “AMEE Consensus Statement: Planetary health and education for sustainable healthcare” [59], “Faculty development and partnership with students to integrate sustainable healthcare into health professions education” [60], “Empowering Patients to Co-Create a Sustainable Healthcare Value” [61].
- Sustainable supply chains: “Integration of a Balanced Scorecard, DEMATEL, and ANP for Measuring the Performance of a Sustainable Healthcare Supply Chain” [62], “The Healthcare Sustainable Supply Chain 4.0: The Circular Economy Transition Conceptual Framework with the Corporate Social Responsibility Mirror” [63], “Managing a sustainable, low carbon supply chain in the English National Health Service: The views of senior managers” [64].

**Figure 5:**
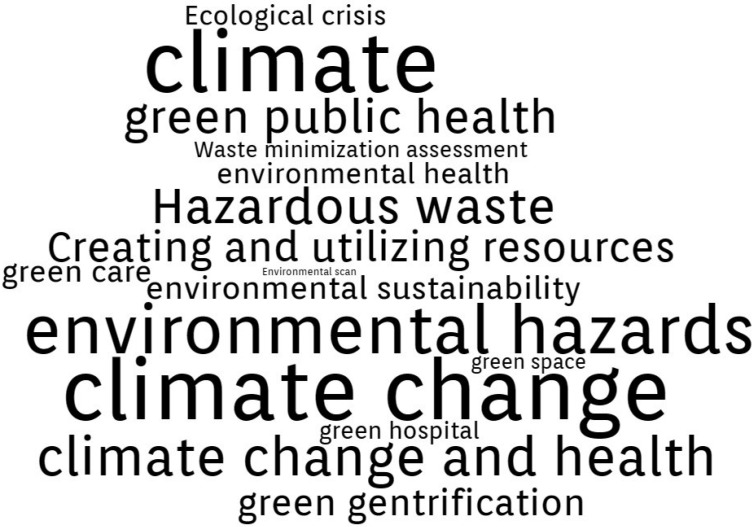
Climate cluster Authors’ Keywords.

Cluster 2, “Climate”, is presented in Figure 5, and includes 19 terms: climate change, climate change and health, environmental hazards, environmental health, environmental health inequalities, Environmental scan, environmental sustainability, green care, green economies, green exercise, green gentrification, green growth strategies, green hospital, green public health, green space, hazardous waste, healthcare waste management, waste minimization assessment, creating and utilizing resources, ecological crisis. In general, the papers referring to this cluster focus on:

- Climate change and health: “Towards Climate Resilient and Environmentally Sustainable Health Care Facilities” [65], “Nurses’ perceptions of climate and environmental issues: a qualitative study” [66], “Impact of a Telemedicine Program on the Reduction in the Emission of Atmospheric Pollutants and Journeys by Road” [67].
- Green health facilities: “Greening healthcare: systematic implementation of environmental programmes in a university teaching hospital” [68], “Residential Greenery: State of the Art and Health-Related Ecosystem Services and Disservices in the City of Berlin” [69], “Using the World Health Organization health system building blocks through survey of healthcare professionals to determine the performance of public healthcare facilities” [70].
- Healthcare resources management: “Healthcare waste generation and management practice in government health centers of Addis Ababa, Ethiopia” [71], “Impact of intervention on healthcare waste management practices in a tertiary care governmental hospital of Nepal” [72], “LCA of Hospital Solid Waste Treatment Alternatives in a Developing Country: The Case of District Swat, Pakistan” [73].

**Figure 6:**
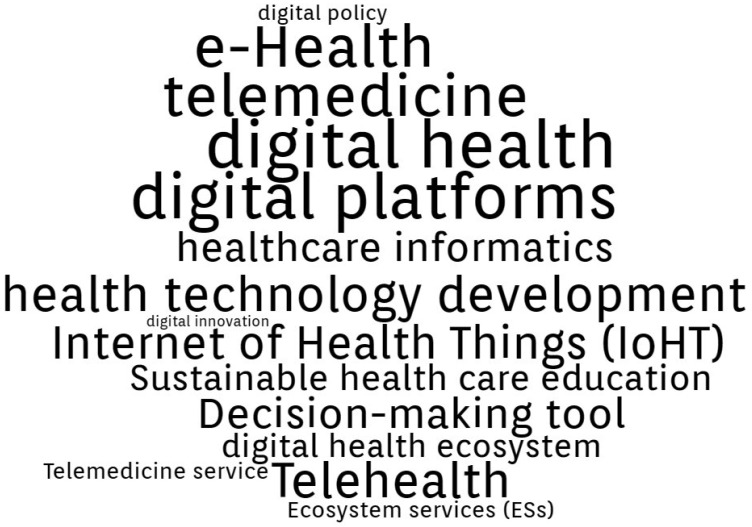
Digital Transformation cluster Authors’ Keywords.

Cluster 3, “Digital transformation”, is presented in Figure 6, and includes 17 terms: digital innovation, digital platforms, digital policy, Internet of Health Things (IoHT), telemedicine, decision support, decision-making, decision-making tool, digital health, digital health ecosystem, telehealth, telemedicine, telemedicine service, ecosystem services (ESs), digital dentistry, health technology development, healthcare informatics. New technology has numerous applications in healthcare allowing for lowering medical costs, upgrading the quality and efficiency of medical procedures, improving healthcare pathways, and giving better control over resource management. All these factors in the healthcare sector contribute to the implementation of sustainable development as promoted and recommended by the United Nations. In general, the papers referring to this cluster focus on:

- Digital innovations and policy for healthcare: “Sustainable Value Co-Creation and Digital Health: The Case of Trentino eHealth Ecosystem” [74], “Engagement in Healthcare Systems: Adopting Digital Tools for a Sustainable Approach” [75], “Pursuing Sustainability for Healthcare through Digital Platforms” [76].
- Decision making support: “Sustainability of knowledge translation interventions in healthcare decision-making: a scoping review” [77], “Sustainability in health care by allocating resources effectively (SHARE) 3: examining how resource allocation decisions are made, implemented and evaluated in a local healthcare setting” [78], “Pinch Analysis as a Quantitative Decision Framework for Determining Gaps in Health Care Delivery Systems” [79].
- Telemedicine: “Improving the Cost-Effectiveness of a Healthcare System for Depressive Disorders by Implementing Telemedicine: A Health Economic Modeling Study” [80], “Impact of a Telemedicine Program on the Reduction in the Emission of Atmospheric Pollutants and Journeys by Road” [67], “Adoption mechanism of telemedicine in underdeveloped country” [81].

### 3.3. Total Number of Publications Per Location

**Figure 7:**
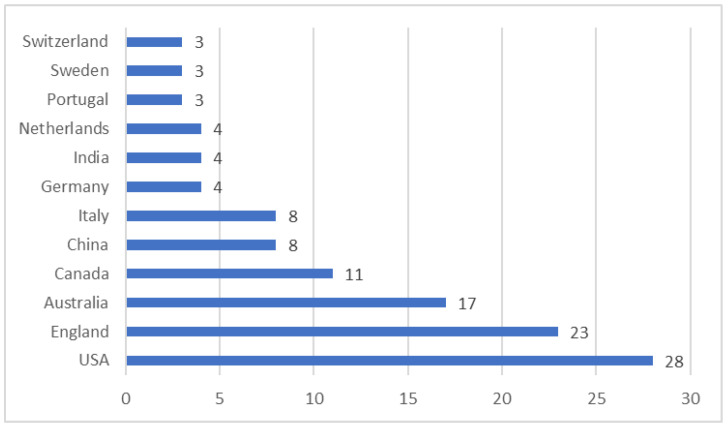
Number of articles per country.

One of the basic criteria for bibliographic analysis is the index of countries that contribute most in this field [82,83]. The twelve leading countries in terms of the number of published articles in the field of the green and sustainable health sector are presented in Figure 7. The country selection criterion for multi-author publications was the country of the corresponding author. Most productive in this field were researchers from the USA, England, and Australia. The leading country in the European Union was Italy.

### 3.4. Total Number of Publications Per Journal

**Figure 8:**
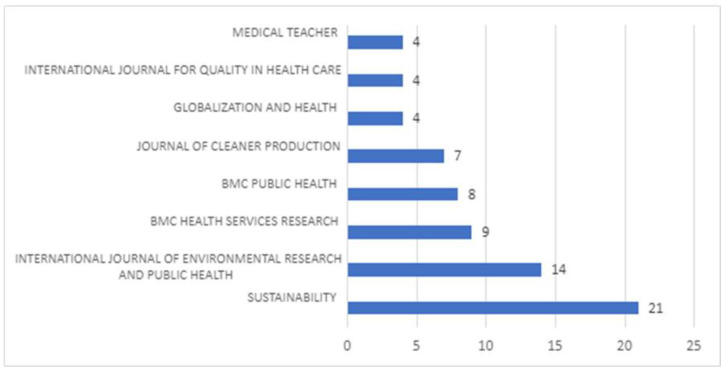
Number of papers per journal.

The 144 articles selected were published in 66 different journals. The Impact Factor (IF) of the journals ranged from 202.731 for The Lancet to 0.863 for Quality in Ageing and Older Adults. Out of 66 scientific journals, we identified seven which are the most popular among researchers (Figure 8). The remaining 89% of journals only occasionally publish articles about eco-health sustainability. 

The most popular journal, Sustainability, is published by MDPI. It is an international, cross-disciplinary, scholarly, peer-reviewed, and open-access journal on the environmental, cultural, economic, and social sustainability of human beings. The Impact Factor of this journal is 3.889. The second-most popular journal is the International Journal of Environmental Research and Public Health, which an interdisciplinary, peer-reviewed, open access journal published semimonthly online by MDPI. It covers Environmental Sciences and Engineering, Public Health, Environmental Health, Occupational Hygiene, Health Economic, and Global Health Research. The Impact Factor of this journal is 4.614.

Further, we highlight the three BMC journals. These are Health Research Policy and Systems, which covers all aspects of the organisation and use of health research, including agenda setting, building health research capacity, and how research as a whole benefits decision makers, practitioners in health and related fields, and society at large, and has an impact factor of 4.139; BMC Public Health journal, with a special focus on the social determinants of health, the environmental, behavioral, and occupational correlates of health and disease, and the impact of health policies, practices and interventions on the community, which has an impact factor of 4.135; and Globalization and Health, a transdisciplinary journal that publishes papers on how globalization processes affect health through their impacts on health systems and the social, economic, commercial, and political determinants of health. The focus of this journal is on policy, systems, technological, organizational, clinical, community and individual perspectives, and it has an impact factor of 10.401.

Similarly impactful is the Journal of Cleaner Production, which serves as a platform for addressing and discussing theoretical and practical cleaner production, encompassing environmental, and sustainability issues in corporations, governments, education institutions, regions, and societies, with an impact factor of 11.072.

Figure 7 covers the International Journal for Quality in Health Care (IJQHC), an interdisciplinary journal in the field of health services research, health care evaluation, policy, health economics, quality improvement, management, and clinical research focused on the quality and safety of care, with an impact factor of 2.257, as well as Medical Teacher, the official journal of the Association for Medical Education in Europe (AMEE). This international journal publishes research on medical education, including the developments in teaching approaches and methods, and has an impact factor of 4.277.

### 3.5. Total Number of Citations Per Paper

**Figure 9:**
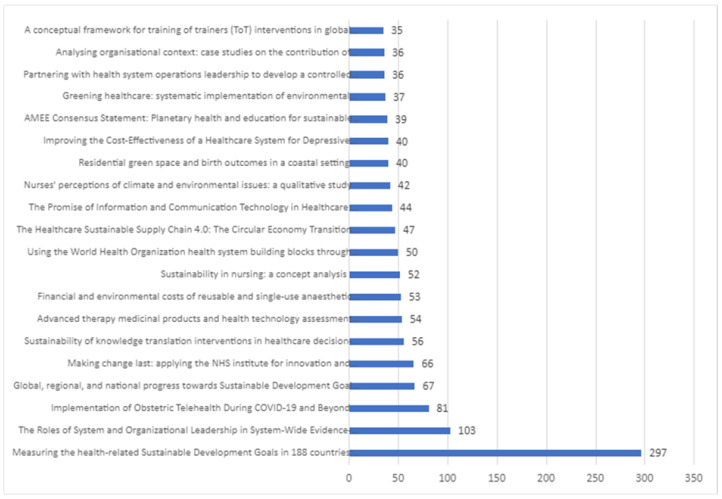
The total number of citations per paper.

The most cited article has at least twice as many citations as any other article in the field (Figure 9).

The most cited article was published in the Lancet in 2015 by researchers from the USA. The title of the article is “Measuring the health-related Sustainable Development Goals in 188 countries: a baseline analysis from the Global Burden of Disease Study 2015” [84]. The research focuses on analysis of 33 health-related SDG indicators based on the Global Burden of Diseases, Injuries, and Risk Factors Study 2015. The indicators have an impact on sustainable or pro-environmental behavior. The objective of the study was to meet a core dimension of SDG Goal 3, aiming to “ensure healthy lives and promote wellbeing for all at all ages”.

Second is the article “The Roles of System and Organizational Leadership in System-Wide Evidence-Based Intervention Sustainment: A Mixed-Method Study” [85], published in the Administration and Policy in Mental Health and Mental Health Services Research in 2016 by an international team from Asia, the USA, and Europe. The article highlights the role of positive sustainable leadership in positively contributing to the implementation of the sustainable development goals in the context of public health. 

Third is the article “Implementation of Obstetric Telehealth During COVID-19 and Beyond” [47], published in the Maternal and Child health Journal (Springer) in 2020 by an American research team, which concludes that “due to the COVID-19 pandemic, implementation of telehealth and telehealth have become crucial to ensure the safe and effective delivery of obstetric care”.

The fourth most cited article is entitled “Global, regional, and national progress towards Sustainable Development Goal 3.2 for neonatal and child health: all-cause and cause-specific mortality findings from the Global Burden of Disease Study 2019” [86], and was published in the Lancet in 2021 by the GBD 2019 Under-5 Mortality Collaborators. It reveals that global child mortality declined by almost half between 2000 and 2019, although progress remains slower in neonates, and 65 (32%) of 204 countries, mostly in sub-Saharan Africa and southern Asia, are not on track to meet either SDG 3.2 target by 2030.

The fifth most-cited article is entitled “Making change last: applying the NHS institute for innovation and improvement sustainability model to healthcare improvement” [87], and was published in Implementation Science in 2013 by a UK research team. The paper states that the Sustainability Model presented by authors is an important attempt to enable teams to systematically consider determinants of sustainability, provide timely data to assess progress, and prompt action to create conditions for sustained practice. Tools such as these need to be tested in healthcare settings to assess strengths and weaknesses and their findings disseminated to aid development.

### 3.6. Most Popular Subject Categories

**Figure 10:**
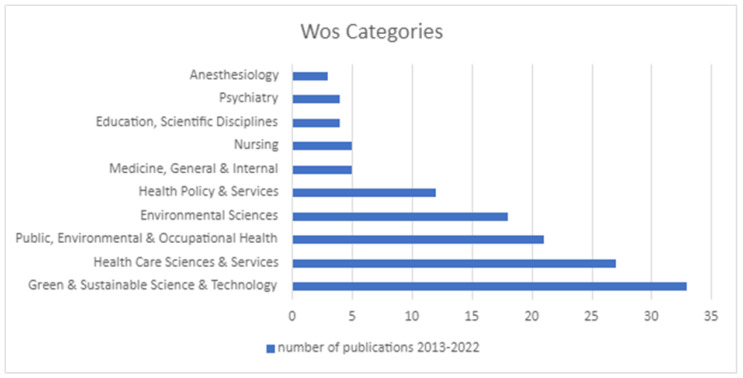
The most popular subject categories.

The final stage of the bibliographic analysis was focused on the most popular WOS subject categories in which the sample papers were published. As presented in Figure 10, the green and sustainable healthcare papers most often related to the categories of Green and Sustainable Science and Technology, Health Care Science and Services, and Public, Environmental, and Occupational Services. It is worth highlighting that green and sustainable papers are popular among professional healthcare categories such as Psychiatry, Anesthesiology, and Nursing as well. The presence of green and sustainable healthcare research topics in educational categories can be seen as a good signal, as everything starts with attitude, knowledge, and skills. 

## 4. Discussion and Conclusions

The problem of sustainable development has been widely discussed in the health sector since the United Nations Conference on the Human Environment in Stockholm in 1972 [1]. On the one hand, this sector is essential to the achievement of the United Nations Sustainable Development Goal (UNSDG) “Good health and well-being” and related goals [9]. At the same time, healthcare, including pharmaceuticals, is responsible for 4.4% of global greenhouse gas emissions [13]. Therefore, green and sustainable development of the health sector is a highly complex, integrated, and interconnected phenomenon. In the last decade, the demand for green healthcare has increased and become more urgent, as health care facilities, in particular hospitals, are organizations that require large amounts of resources for medical services (such as water, electricity, gas, and food), and generate both medical and hazardous waste. Therefore, the green transformation of this sector is crucial to achieving climate and sustainable development goals.

A special role in the green transformation of the health sector is played by scientific research disseminated in the form of scientific publications. Researchers around the world diagnose the current state of affairs, disseminate good practices, develop models, frameworks, and tools, and indicate future directions of development [88]. Thus, the aim of this paper was to present the current state of the art and future research scenarios in the field of green and sustainable healthcare. The authors performed a literature review supplemented by a bibliometric analysis of scientific publications on green and sustainable healthcare published in 2013–2022 using the WOSCC database. A total of 144 papers were included in the final analysis and categorized into eight fields, including author(s), title, published year, country, journal, scientific category, and paper citations. 

As a result of the bibliographic analysis, it was found that the concept of green and sustainable health care is becoming more and more popular among researchers, especially during the last three years. As a result of our systemic review, we can state that the interests of researchers are encapsulated in three thematic clusters: the first (general) focuses on sustainable development in the health sector; the second (climate) focuses on the impact of health sector organizations on the climate or wider environment; and the third (digital transformation) focuses on new technologies applied in healthcare that can support the sustainable development of the sector.

Taking into account the other analyzed indicators, it can be concluded that most research work is concentrated in English-speaking countries such as the USA, UK, Australia and Canada in several interdisciplinary journals, mainly from the publishing houses MDPI, Elsevier, and BMC, and is related to several scientific categories consistent with the thematic clusters identified earlier, namely, technology, environment, health systems, and professions.

The analysis of the publications selected for review revealed blank spots in the issues raised by researchers. For example, regarding resource management, the focus lies mostly on waste management. Little or no research was found within the scope of other green elements of the health care system, such as energy management, water management, transportation, and food, as well as medicines and other chemicals used in this sector. 

There is a lack of work devoted to green leadership in the health sector, which translates into continuing insufficient awareness in the health sector community regarding the negative impact that the sector has on the natural environment and possible steps to be taken to reduce or completely eliminate this impact. Although, as discussed in the introduction, there are numerous international, national and professional initiatives for the green and sustainable recovery sector around the world, these represent only a drop in the ocean of needs, although the intensification of research in this area may lead and guide practice. Thus, the authors recommend that researchers undertake the following research agenda:

1. Development of comprehensive green healthcare assessment tools and maturity models for green and sustainable healthcare organizations to support a holistic transformation of the healthcare sector. This will make it easier for decision makers to make strategic decisions. In addition, such tools and models allow for objective comparison of the level of transformation progress of individual organizations and entire health systems.
2. Further development of the concept of green hospitals and the related evaluation standards, which may be the basis for certification of such facilities. Systematic updating of concepts allows us to capture the latest and most effective process and product solutions and innovations.
3. Diagnosing the use of water and energy resources in the health sector, defining good practices, and proposing paths to optimize the consumption of these resources, as well as initiating innovations in this area.
4. Analyzing and shaping mobility behavior across the entire health sector chain and developing policies and good solutions (especially technological/digital) to minimize the impact of the sector on climate change.
5. Assessing waste management (especially hazardous and medical waste) and developing good practices to minimize harmfulness and waste generation and maximize waste recycling.
6. Exploring current and potential solutions in the area of food systems in order to reduce the environmental footprint of hospitals while shaping healthy eating habits of patients and staff.

The study presented here has a number of limitations. First, the researchers focused on a single database (WOSCC); in the future, it is advisable to use the resources of other databases such as Scopus, PubMed, Cochrane, or EMBAS as well. Moreover, only English-language publications were selected for the analysis. In the future, it would be worthwhile to examine publications on green and sustainable health care in other languages in order to assess their consistency with or differences from the main English-language research stream. The analysis presented here is quantitative, and in subsequent studies it would be well worth supplementing with a qualitative content analysis, which would allow for an overview of the available definitions, models, tools, and measures. It would be most useful to analyze publications in terms of their research methodology and to review the good practices presented in selected publications.

## Data Availability

All data relevant to this work are included in the article or are uploaded as Supplementary Information.

## Supplementary Materials

The following supporting information can be downloaded at: https://www.mdpi.com/article/10.3390/ijerph20020908/s1, File S1: The database of final 144 records included in review (excel). File S2: The database of 836 records included in the VOSviewer visualisation (TXT format). File S3: he database of 836 records included in the VOSviewer visualisation (RIS format).
